# DBGCN: Dual-branch Graph Convolutional Network for organ instance inference on sparsely labeled 3D plant data

**DOI:** 10.1016/j.plaphe.2026.100188

**Published:** 2026-03-09

**Authors:** Dawei Li, Zhaoyi Zhou, Si Yang, Weiliang Wen

**Affiliations:** a School of Information and Intelligent Science, Donghua University, Shanghai, 201620, China; b State Key Laboratory of Advanced Fiber Materials (SKLAFM), Donghua University, Shanghai, 201620, China; c Engineering Research Center of Digitized Textile & Fashion Technology, Ministry of Education, Donghua University, Shanghai, 201620, China; d Information Technology Research Center, Beijing Academy of Agriculture and Forestry Sciences, Beijing, 100097, China; e Beijing Key Lab of Digital Plant, National Engineering Research Center for Information Technology in Agriculture, Beijing, 100097, China

**Keywords:** Organ instance segmentation, Plant point cloud, Plant phenotyping, Transductive learning, Dual-branch graph convolutional network

## Abstract

3D crop phenotyping technology provides critical support for screening morphology-related plant genes and identification of germplasm resource. Organ segmentation or recognition is the first key step in 3D crop phenotyping, where inductive deep learning currently dominates as the mainstream methodology. However, the high requirement for data annotation in inductive learning paradigm has transformed the manual data labeling into a labor-intensive task, thereby in turn restricting the progress of inductive learning. This problem has inspired us to leverage Graph Neural Networks (GNNs) as the transductive learning tool to directly segment organs on sparsely annotated crop point clouds. We propose a Dual-branch Graph Convolutional Network (DBGCN) that only requires sparse labels to perform organ instance inference directly on plant point clouds that have featureless point features. Different from existing graph-based networks, DBGCN not only carries out the static-feature-space graph convolutions that are good at mining and aggregating on local information on the point cloud, but also incorporates dynamic graph convolutions that captures the potential changes of the graph manifold in deep feature space. Extensive experiments prove that the fusion of two types of graph feature convolutions brings a high node (point) classification accuracy, outperforming mainstream GNNs and even several popular inductive deep architectures. On the PlantNet sub-dataset, DBGCN achieves an mAcc (mean accuracy of node classification) of 93.00% under 1.95% manual annotation ratio. On the Soybean-MVS sub-dataset, DBGCN achieves an mAcc of 91.05% under 4.88% manual annotation ratio. Furthermore, our DBGCN not only works well on crop 3D data but can also serve other applications such as the segmentation of point cloud data for large-scale street view. Our dataset and code can be found at https://github.com/chinazhouzhaoyi/DBGCN/tree/master/

## 1 Introduction

Food security remains a significant global issue for human development due to the impacts of climate change and limited arable land resources [1]. Therefore, the selection of high-yield crop varieties has long been an important task in modern agricultural research [2]. Plant phenotyping plays a vital role in identifying high-yield functional genes and hence promoting modern breeding. Phenotypes are defined as the physical, physiological, and biochemical features and traits resulting from the interaction between genes and the environment, including components such as morphological structures, developmental processes, and stress resistance [3,4]. With the advancement of 3D imaging and sensing technologies, 3D crop phenotyping is becoming increasingly popular because it circumvents the issues of visual overlapping and occlusion [5] that are quite common in traditional image-based phenotyping. Nowadays, researchers are contactlessly acquiring 3D point cloud data of plants [6,7] with continuously reducing cost and rapidly increasing accuracy at the same time. Therefore, automatic segmentation/detection of organs from those 3D data naturally becomes the urgent task that follows [8]. Based on segmented organ instances, researchers are then able to extract accurate organ-level phenotypic traits, such as leaf area, leaf count, stem length, fruit size, etc.

**Fig. 1:**
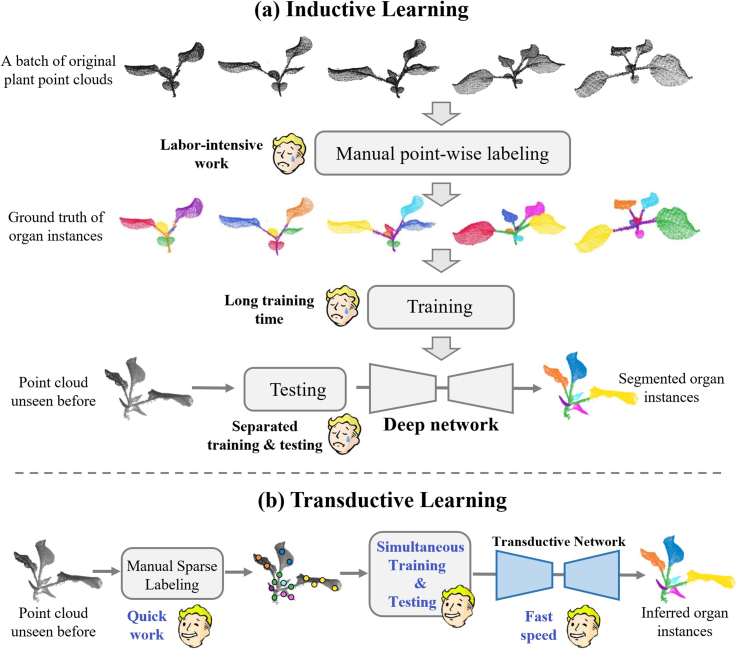
The difference between the traditional inductive learning and the new transductive learning paradigm for organ instance segmentation task on crop point cloud. (a) is the flowchart of the traditional inductive learning paradigm in which deep networks require a large number of manually labeled crop point clouds for training. (b) is the flowchart of the transductive learning paradigm, in which only a sparsely labeled point cloud is required, and training and testing are done at the same time.

Currently, the inductive learning paradigm based on Deep Neural Networks (DNN) dominates the methodological landscape for segmenting plant point clouds. Enlightened by U-Net-like architectures, the PointNet++ [9] family has been widely employed for crop point cloud classification [10], organ segmentation [11,12], and biomass regression tasks [13]. A standard inductive learning pipeline for organ instance segmentation from point clouds is illustrated in Fig. 1(a). After training on large-scale datasets that contain thousands of crop samples [14,15], the inductive deep network can segment plant point clouds that have never seen before. Although inductive learning has become the mainstream paradigm for 3D point cloud deep learning systems, it puts two strong requirements on data—(i) the training samples should be rich in number and diversified in morphological structure, and (ii) the dataset usually requires point-level manual annotation. These requirements turn the human annotation of point cloud data into a labor-intensive and time-consuming process. The challenge is even amplified by practical situations in agricultural scenarios—such as missing data and noise. The construction of high-quality annotated 3D datasets thus becomes an obvious bottleneck in plant phenotyping, which in turn restricts the application of modern inductive learning paradigm. And this motivates us to turn to other learning approaches for automatic organ segmentation on 3D plant data.

Transductive learning (Fig. 1(b)) is a powerful paradigm that commonly takes the shape of semi-supervised learning methods to enable simultaneous training and inference on individual samples with sparse annotations. However, in order to ensure the accuracy of point-level inference, it is necessary to mine the latent correlations across all points, such as the local neighborhood connectivity in space, inter-point feature distances or similarities. Therefore, transductive learning approaches are particularly well-suited for graph-structured data representations. Recent advances in Graph Neural Networks (GNNs) have significantly simplified the deployment of semi-supervised-based transductive learning frameworks that classify graph nodes or partition a graph into substructures through specialized convolutions and feature aggregation mechanisms. In the scope of GNNs, the Graph Convolutional Network (GCN) and their variants [[16], [17], [18]] were especially welcomed among researchers because their evident advantages including sparse and quick annotation, no need for pretraining, and fast inference speed. Moreover, 3D point clouds can be naturally represented with graphs, in which points are treated as vertices while the relationship between two near points is treated as an edge. This graph representation of point cloud motivates us to leverage GCN-based transductive learning for direct organ instance segmentation on sparsely annotated plant point clouds. However, existing GCNs are made for graphs with rich feature inputs from the domains such as social networks and bioinformatics. The performances of those models on 3D point clouds whose input point feature vectors are “featureless” (each colorless point is represented by a “short” 3-dimensional XYZ vector) are either unstable or unclear. Despite the theoretical potential, the graph-based transductive learning paradigm remains largely underexplored in the 3D plant phenotyping field. We hope to overcome the limitations of inductive learning on real agricultural scenarios that are filled with incomplete 3D data and extremely sparse annotation, by taking the first step on designing a novel transductive neural architecture.

This paper proposes a new transductive Dual-branch Graph Convolutional Network (DBGCN) for organ instance inference on plant point cloud; the contributions are listed as follows:

(i) DBGCN can directly perform organ instance segmentation on a single point cloud with only sparse organ labels. Its transductive framework not only significantly reduces the workload of manual annotations but also smoothly runs on small crop point cloud datasets that are unable to be trained by inductive learning methods.
(ii) In order to transcend the existing GCNs for transductive learning on featureless plant point clouds, our DBGCN makes two substantial improvements—input feature engineering and diversified graph convolutions. For input feature engineering, a local similarity encoded from adjacency matrix is combined into the coordinate space to enrich the feature vector of each input point of the graph. In order to enable highly effective feature extraction in a few neural layers, we diversify the graph convolutions by a novel dual-branch graph convolution architecture that absorbs the advantages of the static-feature-space and the dynamic-feature-space graph convolutions.
(iii) The proposed network outperforms multiple mainstream GNNs and are even superior to several popular inductive learning architectures for organ instance segmentation. On a three-species point cloud dataset [19,20], DBGCN achieves 93.00% segmentation accuracy under 1.95% labeling ratio to the total points. On the Soybean-MVS dataset [21] with 4.88% point labeling ratio, DBGCN achieves segmentation accuracy at 91.05%. Our DBGCN even shows potential usefulness on datasets of other fields such as large-scale 3D street view.

The remainder of this paper is structured as follows: section 2 reviews related work in 3D plant phenotyping and GNN methodologies; section 3 elaborates the design of the proposed transductive Dual-branch Graph Convolutional Network; section 4 presents the comparative results between our architecture with state of the art on datasets containing multiple crop species; and section 5 suggests the further optimization of DBGCN. Finally, section 6 concludes the paper.

## 2 Related work

### 2.1 3D plant phenotyping and inductive learning

Morphological and structural phenotypes of plants have received extensive interest in plant phenomics research. Certain plants exhibit distinctive but important morphological traits, such as the number of tillers on rice plant [22], the height of maize plant [23], and the number of grains and pods per plant on soybean plants [24]. These traits serve as foundations for crop digital twin realization and variety selection. 3D point cloud data presents the true spatial structure of plants, and overcomes the occlusion issue and perspective distance errors that are common in 2D imagery [25]. With advancements in 3D imaging and sensing, the performance-price ratio of 3D scanning devices has been increasing rapidly, opening the 3D era for plant phenotyping. Techniques such as LiDAR [26], multi-view imaging [27], and 3D scene reconstruction [28] have been employed to acquire 3D plant point cloud data and extract phenotypic parameters (e.g., plant main stem height and leaf area) to understand plant growth and development. Based on high-precision point clouds, traditional 3D plant phenotyping methods predominantly adopt unsupervised algorithms to segment organs or plant parts. To separate the maize seedlings from the reconstructed 3D field, Xu et al. [29] proposed a novel method for detecting maize seedlings using features derived from Terrestrial Laser Scanning (TLS) and camera data, which successfully separates maize seedlings from soil. Sun et al. [30] designed a density-based clustering method for segmenting reconstructed cotton point clouds from multi-view stereo for yield prediction. To accelerate the phenotyping process in sugar beet breeding, Xiao et al. [31] developed an automated pipeline for processing sugar beet point clouds to extract phenotypic traits such as plant height, maximum canopy area, convex hull volume, total leaf area, and individual leaf length.

Purely unsupervised segmentation algorithms have limited capability on segmenting overlapping leaves. To address the instance segmentation issue in overlapping canopy clusters, researchers turned to the integration of multiple algorithms. Liu et al. [32] used a region-growing algorithm based on Multi-scale Tensor Voting Method (MSTVM) to segment preprocessed sugar beet point clouds, generating independent and overlapping leaf sets. Then, they applied the Surface Boundary Filter (SBF) [33] to segment overlapping leaves into individual leaves. Unlike the aforementioned methods that use local geometric features of point clouds for organ instance segmentation, Mirande et al. [34] proposed a three-stage organ instance segmentation method based on graphs. They first performed preliminary geometric clustering by computing both local geometric and spectral features on the neighborhood graph of points, then refined the overall consistency of segmentation by using the quotient graph of each detected organ with its adjacent organs. Recently, Wang et al. [35] also noticed the importance of integrating the global structural features with local morphological features in point clouds.

Unsupervised 3D crop phenotyping pipelines are composed of complicated steps [36,37] with a number of parameters to be set, resulting in poor generalization across species. Inductive learning, being the most common and intuitive machine learning paradigm, is a classical paradigm that accompanies the evolvement of neural networks. In recent years, the rapid development of inductive deep learning has made the data-driven methods to be the major way of processing high-precision, large-scale, and high-throughput crop point cloud data [38]. These networks are trained with large-scale training sets for classification and segmentation tasks, and the trained networks can perform well on data never seen before. This explains the dominance of inductive learning in current deep networks for processing plant point clouds. To tackle the unordered and non-uniform data distribution in point clouds, the classical inductive deep network PointNet [39] employed multi-layer 1D convolutions for feature extraction, followed by permutation-invariant pooling operations to derive global feature vectors. Its hierarchical variant, PointNet++ [9], introduced progressive point sampling and neighborhood aggregation mechanisms to abstract high-level semantic information across neural layers. Extending the concept of PointNet++, DGCNN [40] designed EdgeConv operations on dynamic graph feature spaces for neighborhood-related feature extraction, and enhanced segmentation performance on point clouds. The PointNet architecture family outperformed traditional unsupervised methods for the organ-level semantic segmentation task on crops such as maize [12], tomato [41], and sorghum [42].

However, crop point clouds exhibit higher irregularity compared to other types of objects (e.g., furniture, vehicles). Transferring generic point cloud segmentation networks directly to the crop datasets will probably cause a drop in performance; therefore, specialized networks for crop point clouds have been emerging. For instance, Gong et al. [43] developed Panicle-3D, a network tailored for rice panicle segmentation. Panicle-3D mitigates feature loss during down-sampling, which helps to better separate panicles from stems. Wang et al. [44] proposed a top-down recursive decomposition network for lettuce instance segmentation, leveraging PointNet++ as feature extraction backbone. Guo et al. [45] proposed a segmentation method combining deep learning and clustering algorithms to enhance the organ-level segmentation accuracy of cabbage point clouds. First, they utilized attention mechanism for semantic segmentation of the stem and leaves, and then employed DBSCAN for leaf instance clustering. Liu et al. [46] proposed a wheat spike segmentation method combining Kernel Prediction Convolutional Neural Network (KP-CNN), density-based spatial clustering, and Laplacian-based region growing techniques to segment wheat spikes from field-collected Lidar data and calculated traits. Peng et al. [47] presented SqueezeNet using time-series point cloud sequences and as input, and reported SOTA performance on the Pheno4D benchmark [48].

Despite these achievements, directly output organ instances from a network is still a difficult task. This limitation has spurred the development of inductive dual-function architectures capable of segmenting organ semantics and organ instances simultaneously [14,15,49,50]. Jin et al. [49] proposed a Voxel-based Convolutional Neural Network (VCNN) for stem-leaf classification of maize point clouds. Du et al. [50] presented PST-Net, which was specially designed to conduct silique instance segmentation for rapeseed point clouds. Li et al. developed PlantNet [14] and PSegNet [15], both are dual-function architectures that achieved organ semantic and instance segmentation across point clouds of multiple crop species.

Though we have already witnessed notable advancements gained by inductive deep learning frameworks in 3D crop phenotyping, these methods impose two strict requirements on data—(i) the training samples should be rich in number and diversified in morphological structure, and (ii) the dataset usually requires point-level manual annotation. These requirements turn the human annotation of point cloud data into a labor-intensive and time-consuming work, and human annotators are prone to errors in the tedious and boring annotation process. In practical agricultural environments, the acquired 3D plant data often suffers from high structural complexity, insufficient data, and intense noise levels, which all hinder the preparation of high-quality annotated datasets; and the quality of inductive model training is dependent on good annotation. The restriction on agricultural 3D data drives us to seek another path beyond the traditional inductive learning paradigm.

### 2.2 Semi-supervised Graph Neural Networks

In contrast to the inductive learning paradigm, transductive learning typically operates within a semi-supervised framework, requiring only partial or sparse labeling on a single data sample to simultaneously complete training and inference for the rest unlabeled points. Perhaps the most well-known traditional semi-supervised algorithm is the semi-supervised KNN. If one wants to propagate labels from the point set $V_{L}$ to a nearby set $V_{U}$, the semi-supervised KNN extracts an unlabeled point from $V_{U}$ then computes the nearest K points in $V_{L}$ and carries out voting. The unlabeled point is then assigned the label that won in voting, and this labeled point was finally moved from $V_{U}$ to $V_{L}$. However, traditional semi-supervised methods only perform well on data containing either clusters that obey a similar probability distribution, or clusters that have little (or no) overlap; and both conditions are not satisfied in large-scale graph datasets in the real world. Common graph data is usually non-Euclidean formed; its common categories include knowledge graphs, social networks, molecular structures [51], and most important of all—point clouds. Achieving accurate point-level inference on graphs requires mining latent correlations across all data points, such as the local neighborhood connectivity in space, inter-point feature distances or similarities. Therefore, graph-based transductive learning approaches seem to be a good solution for graph representations that lack translational invariance and stable geometric structure [52].

Due to the absence of standard coordinates, directly applying Euclidean convolution operators (e.g., CNNs) to graph learning tasks usually leads to poor performance because of the failure to properly utilize the topological information within the graph. To address the convolution problem on graph data, early Graph Neural Networks (GNNs) emerged as specialized architectures for node classification and graph partitioning through tailor-made convolutions and feature aggregation strategies. Bruna et al. [53] pioneered spectral-domain graph representation learning, they initially converted the graph data in the spatial domain to the spectral domain for filtering, making the graph representation learning feasible. Defferrard et al. [54] designed fast local convolutional filters based on Chebyshev polynomials to stably learn local combinatory features on graphs. Niepert et al. [55] further proposed a universal graph convolutional framework that subtly applies traditional image convolution kernels to locally connected nodes for feature extraction. Hamilton et al. [56] introduced GraphSage, which learns neighborhood aggregation functions for nodes in randomly split subgraphs of large graphs, thereby enabling inductive inference on nodes never seen or entirely new subgraphs within a large-scale graph. The emergence of Graph Convolutional Networks (GCN) [16] removed the final obstacle to conduct transductive learning on graph data, marking a milestone in the field. GCNs take initial node feature and adjacency matrix as inputs and can quickly propagate sparse node labels to other unlabeled nodes using just a few layers of neurons. Since its proposal, many GCN variants have been developed, and they can be broadly categorized into: 1-hop (the first-order neighbor processing) methods [17,[57], [58], [59]], Multi-hop (multi-order neighbor processing) methods [[60], [61], [62]], and Graph Data Augmentation which performs view transformation or data perturbation on graphs to enhance network performance [18,63,64].

3D plant point clouds can be naturally represented using graphs, where points in the point cloud serve as graph vertices, and the relationships among neighboring points (such as distance, color similarity, and local geometric similarity) act as edges. It is natural to consider utilizing transductive GCN to perform direct organ instance segmentation/annotation on sparsely labeled crop point clouds. However, crop canopies are usually crowed with a large number of organs (e.g., leaves, petioles, and branches). Frequently observed occlusions and overlapping among organs inside canopies may easily result in inaccurate message passing and false label propagations in existing GCN variants. Furthermore, each input point from a colorless point cloud has an only 3-dimensional feature vector—XYZ coordinates; while the point input from a color LiDAR is a short 6-dimensional feature vector—XYZRGB. This means the input features from a plant point cloud are really “featureless” comparing to the graphs collected from domains such as social networks and bioinformatics, where the node feature is rich. Though the existing GCN variants are good players on graphs with rich input features; they may not align with featureless graphs. Regrettably, except for only a handful of studies [65,66] attempting to apply GCNs to semi-automatic organ annotation of crop point clouds, the transductive GCN learning paradigm has not received attentions that it should have in the field of 3D crop phenotyping. The above facts strengthen our determination to design a new graph learning model for organ instance inference on plant point clouds.

## 3 Method

### 3.1 Task statement

The task of this paper is to perform organ segmentation (or inference) on crop point cloud with sparsely annotated organ labels (Fig. 1(b)), by automatically propagating the labels to all points in the point cloud. Since the point cloud only has sparse labels, this is a typical semi-supervised learning problem. A crop point cloud containing N points can be converted into a homogeneous graph G=(V,E), where V is the vertex set consisting of N nodes, and each node ν∈V has a feature vector $x_{\nu }\in {\mathbb{R}}^{d}$. E is the set of undirected edges that captures all connections among the nodes, and it is generally transformed into an adjacency matrix A. Due to the fact that each input point from a colorless point cloud has an only 3-dimensional (XYZ) feature vector, d equals to 3 and this creates a “featureless” input that lacks further information. In the vertex set, the annotated nodes form a subset with Ground Truth (GT) label information. This GT subset is denoted as $V_{GT}$ (the training set), providing supervised signals in learning. The remaining subset of unlabeled vertices is denoted as $V_{raw}$ (the testing set). Typically, in a sparsely labeled crop point cloud, the proportion of $V_{GT}$ in V does not exceed 10%. We aim to design a novel transductive graph convolutional network to propagate the organ labels from $V_{GT}$ to $V_{raw}$ with a higher accuracy than any other graph-based networks currently known.

### 3.2 Architecture of dual-branch graph convolutional network

**Fig. 2:**
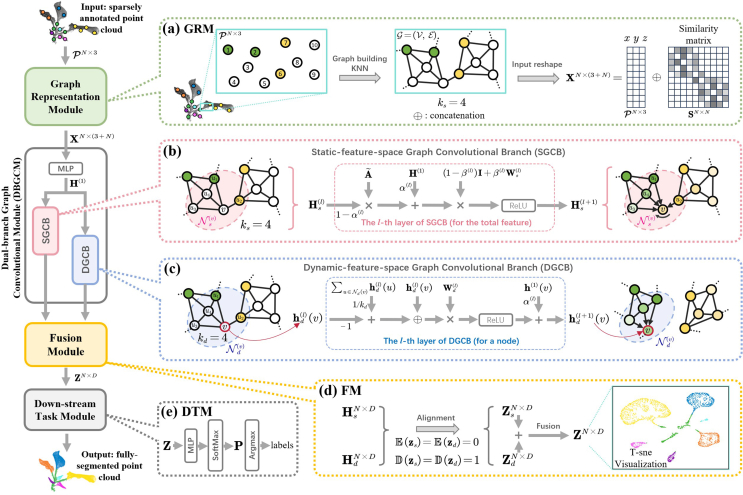
The Dual-branch Graph Convolutional Network (DBGCN) framework. (a) is the Graph Representation Module (GRM), (b) is the Static-feature-space Graph Convolutional Branch (SGCB), and (c) is the Dynamic-feature-space Graph Convolutional Branch (DGCB). Together, (b) and (c) form the Dual-Branch Graph Convolution Module (DBGCM). (d) represents the feature Fusion Module (FM), and (e) is the Downstream Task Module (DTM) for organ label propagation.

We propose a novel transductive Dual-branch Graph Convolutional Network (DBGCN), whose framework is shown in Fig. 2. The DBGCN architecture can be mainly divided into four modules. The first module is the Graph Representation Module (GRM) which converts crop point clouds into inputs that can be processed by graph convolutions and also merges node coordinates with local neighborhood information to create richer input features. The second module is the Dual-Branch Graph Convolutional Module (DBGCM), which performs graph convolutions on a static feature graph and a dynamic feature graph on two branches, respectively. The third module is the Fusion Module (FM), which integrates the dynamic features from nodes and the static features from global graph structure. The fourth module is the Downstream Task Module (DTM), which is designed for semi-supervised node label propagation. Each module will be elaborated in detail below.

### 3.3 Graph Representation Module (GRM)

The first unique processing stage of our DBGCN is the input feature engineering, which is carried out within a Graph Representation Module to increase the vector length (information richness) of each input node. The GRM (Fig. 2(a)) consists of two steps—graph building and input reshaping. In graph building, an undirected graph G=(V,E) is constructed based on the crop point cloud that contains only three-dimensional coordinate information $P^{N\times 3}$. KNN is used to establish the 1-hop neighborhood relationships across all nodes, producing a sparse adjacency matrix $A^{N\times N}$. The number of the nearest neighbors set as $k_{s}$ in KNN directly determines the 1-hop neighborhood size $N_{s}\left(v\right)$ for each node ν. The importance of node neighborhoods for convolutional calculations has been widely discussed [59], and following this line, the sparse adjacency matrix A is first encoded into a similarity matrix $S\in R^{N\times N}$. Unlike the adjacency matrix A which assigns a value of 1 to all connected edges, the similarity matrix S calculates each element value based on the normalized Gaussian smoothing mapping of the distance between each node ν and its neighbors $N_{s}\left(v\right)$. The equation for calculating the similarity matrix S is as follows:

(1) $${\left[S\right]}_{v,u}=\left\{ \begin{array}{c} \frac{\exp\left(-d_{vu}/kernel\right)}{\sum_{u}\exp\left(-d_{vu}/kernel\right)},\;u\in N_{s}\left(v\right)\\ 0,\;\;\;\;\;\;\;\;\;\;\;\;\;\;\;\;\;\;\;\;\;\;\;\;\;\;\;\;\;\;\;\;\;\;\;\;\;u\notin N_{s}\left(v\right) \end{array}\right.\text{.}$$

In equation (1), $d_{vu}$ represents the distance from the neighboring node u to the central node v, and kernel is the Gaussian kernel parameter. The similarity matrix S has two advantages over A: (i) it provides a detailed description of the 1-hop neighborhood graph structure; (ii) it reduces noise in the local neighborhood via nonlinear mapping and smoothing.

The second step of GRM performs input reshaping. Our DBGCN requires two types of inputs—the node feature and the adjacency matrix. By concatenating the coordinates of all nodes $P^{N\times 3}$ with the similarity matrix S, the node feature input X is obtained as:

(2) $$X^{N\times \left(3+N\right)}=P⊕S\text{.}$$

In each row of X, the local neighborhood information of a point is directly embedded into the node feature.

In subsequent calculations, the adjacency matrix A is required as another input to DBGCN. A standard adjacency matrix does not contain self-loops, which overlooks the importance of a node itself. Therefore, we add an identity matrix I to it, resulting in $\tilde{A}=A+I$. It also should be noted that no further normalization is needed for $\tilde{A}$, as the subsequent graph convolutions can handle it directly.

### 3.4 Dual-branch Graph Convolutional Module (DBGCM)

The second improvement of our DBGCN over existing GCN variants lies in its diversified graph convolutions, and a new Dual-branch Graph Convolution Module (DBGCM) is designed to carry out two types of convolutions at the same time. The module consists of two stages—(i) the feature dimensionality reduction, and (ii) the graph convolution computation. In the feature dimensional reduction stage, to improve computational efficiency, the node feature input X from the previous module (size N×(3+N)) is first concentrated in dimensionalities using a fully-connected Multi-layer Perceptron (MLP) to obtain the initial node feature $H^{\left(1\right)}$ of size N×D:

(3) $$H^{\left(1\right)}=MLP\left(X\right)\text{.}$$

Then, $H^{\left(1\right)}$ becomes the shared node feature input for the next stage—graph convolution computation. The graph convolution computation has a dual-branch structure consists of a Static-feature-space Graph Convolutional Branch (SGCB) and a Dynamic-feature-space Graph Convolutional Branch (DGCB). In SGCB (Fig. 2(b)), we adopt the same strategy as most of the GCN family members, which performs message aggregation using the initially fixed feature neighborhood $\tilde{A}$. This also explains why the branch is named as “static-feature-space”. Specifically, we use GCNII [17] as the backbone, if letting $H_{s}^{\left(l\right)}$ denote the input feature at the l-th layer, the output feature $H_{s}^{\left(l+1\right)}$ of that layer (also the input feature of the next layer) can be expressed in equation (4):

(4) $$H_{s}^{\left(l+1\right)}=\sigma \left(\left(\left(1-\alpha^{\left(l\right)}\right)\tilde{A}H_{s}^{\left(l\right)}+\alpha^{\left(l\right)}H^{\left(1\right)}\right)\left(\left(1-\beta^{\left(l\right)}\right)I+\beta^{\left(l\right)}W_{s}^{\left(l\right)}\right)\right)\text{,}$$

where $\alpha^{\left(l\right)}$ is a hyperparameter controlling the residual connection of the initial feature at the l-th layer, typically set to 0.1. The feature transformation matrix $W_{s}^{\left(l\right)}\in R^{D\times D}$ is a learnable parameter matrix. $\beta^{\left(l\right)}$ is a function controlling the identity mapping at the l-th layer, which can be set to log(λ/l+1), where λ is usually set to 0.5. The function σ(·) represents the ReLU non-linear activation function. To reduce the risk of overfitting, L2-regularization is applied to the parameter matrix $W_{s}^{\left(l\right)}$. It is worth noting that, as the layer goes deeper, the information from shallow layers is adaptively increased through $\beta^{\left(l\right)}$, which helps to preserve the initial global graph structure and hence alleviating the feature smoothing problem in graph convolutions.

Because crop point clouds are inherently homogeneous graphs, SGCB easily realizes semi-supervised label propagation through the neighborhood connectivity in points from crop organs. In addition, SGCB has a low parameter quantity and fast processing speed. However, convolutions on solely static feature space have several drawbacks. Firstly, if the static networks (such as GCNII and SGCB) go deeper, their graph learning capabilities will quickly hit a bottleneck and may even decline. Secondly, at organ instance boundaries such as the junction between a stem and a leaf, the static graph convolution tends to learn shallow geometric features, and finally results in poor semantic discernability for boundary nodes. Thirdly, the static graph convolutions smooth the features of nodes and their neighborhoods, and when the static network deepens, it is inclined to focus on the global structure rather than the local details in the graph. This results in errors in label propagation for small plant organs; for example, the missing buds or incorrect leaf edge labels. Therefore, we design a DGCB that operates in parallel with the SGCB.

Unlike the SGCB that fixes the global feature neighborhood of the entire graph as $\tilde{A}$, the DGCB (as shown in Fig. 2(c)) search KNN on its current feature space to obtain the local neighborhood $N_{d}\left(v\right)$ for each target node v. In each layer of the network, the dynamic graph convolution first computes a feature transformation function $F_{d}\left(\cdot \right)$ to convert the neighborhood features of the target node v into edges with the following form:

(5) $$F_{d}\left(v\right)=e_{uv}^{\left(l\right)}=h_{d}^{\left(l\right)}\left(u\right)-h_{d}^{\left(l\right)}\left(v\right),\;\forall u\in N_{d}\left(v\right)\text{,}$$

where $h_{d}^{\left(l\right)}\left(v\right)$ is a D-dimensional vector representing the input feature vector of the target node v in the l-th layer of DGCB. Node u is a neighbor of v in the current feature layer. Next, local aggregation is performed. To better handle the unordered nature of vertices in graph, a symmetric function G(·) is used to aggregate local information. Hereby, we adopt the arithmetic mean operation as G(·). To incorporate both the target node feature and its dynamic neighborhood, a feature concatenation operation “⊕” is performed. The dynamic local neighborhood feature $m_{d}^{\left(l\right)}\left(v\right)$ of the target node v at layer l is calculated as in equation (6):

(6) $$m_{d}^{\left(l\right)}\left(v\right)=G\left(F_{d}\left(v\right)\right)⊕h_{d}^{\left(l\right)}\left(v\right),\;\;\;\forall u\in N_{d}\left(v\right)\text{.}$$

Finally, the node feature is updated. To reduce the loss of original low-level information, a residual mapping on the initial feature $h^{\left(1\right)}\left(v\right)$ is introduced. The dynamic output node feature of node v at layer l is:

(7) $$h_{d}^{\left(l+1\right)}\left(v\right)=\sigma \left(m_{d}^{\left(l\right)}\left(v\right)\cdot W_{d}^{\left(l\right)}\right)+\alpha^{\left(l\right)}h^{\left(1\right)}\left(v\right)\text{,}$$

where $W_{d}\in R^{2D\times D}$ is a learnable feature transformation matrix. This matrix is similar to the parameter matrix $W_{s}$ used in SGCB, and all nodes in the graph share the same transformation matrix. The setting of $\alpha^{\left(l\right)}$ in equation (7) is the same as in (4). After updating the node features, all node features $h_{d}^{\left(l+1\right)}\left(v\right)$ are stacked to form a feature matrix $H_{d}^{\left(l+1\right)}$. The advantages of DGCB are at least two-fold. Firstly, as the number of layers increases, $N_{d}\left(v\right)$ is not limited to a fixed 1-hop neighborhood, gradually expanding the receptive field. Secondly, it enables label propagation in high-dimensional spaces, which is beneficial for the classification of nodes at boundaries separating different classes.

Different from existing GNNs, our convolutional module contains two types of graph convolutions—the static-feature-space graph convolutions are good at mining and aggregating the local information on the point cloud, while the dynamic graph convolutions capture the potential changes of the graph manifold in deep feature space. The SGCB branch of DBGCM can effectively extract the “static” global point cloud structure and meanwhile the DGCB branch precisely propagates the messages of high-frequency point cloud details in the “dynamic” variations of the feature space. Therefore, this dynamic-static combined module design is beneficial for a thorough understanding of point clouds. Readers are referred to subsection Appendix 7.1 for a detailed discussion on the comparison of the static and the dynamic graph convolutions.

### 3.5 Fusion Module

After processing with SGCB and DGCB, the point cloud graph feature X is transformed into $H_{s}$ and $H_{d}$, respectively. These two types of features are integrated through the Fusion Module (Fig. 2(d)). First, node-level feature alignment is performed on both $H_{s}$ and $H_{d}$. We expect the node features to obey a distribution with zero mean and a variance of 1. The node feature alignment is realized by the following equations:

(8) $$z\left(v\right)=\frac{h\left(v\right)-\overset{D}{\overbrace{\left[E\left(h\left(v\right)\right),\ldots ,E\left(h\left(v\right)\right)\right]}}}{\sqrt{D\left(H\right)+\delta }}⊗\gamma +\xi \text{,}$$

(9) $$E\left(h\left(v\right)\right)=\frac{1}{D}\sum_{j=1}^{D}{\left[H\right]}_{v,j}\text{,}$$

(10) $$D\left(h\left(v\right)\right)=\frac{1}{D}\sum_{j=1}^{D}{\left[{\left[H\right]}_{v,j}-E\left(h\left(v\right)\right)\right]}^{2}\text{,}$$

in which ${\left[H\right]}_{v,j}$ represents the element located at the v-th row and j-th column of the feature map H; E(h(v)) and D(h(v)) are the mean and variance of the node feature of v in H. To avoid divided by zero in equation (8), a small number $\delta =1\cdot e^{-5}$ is added to the denominator. The parameter vectors γ and ξ are learnable D-dimensional parameter vectors used to adjust the feature maps to achieve better fusion results. The notation “⊗” denotes element-wise multiplication. Feature matrices $H_{s}$ and $H_{d}$ are processed through equation (8) to obtain the aligned node feature matrices $Z_{s}$ and $Z_{d}$, respectively. Node feature alignment offers three main benefits—(i) it aligns node features of the two branches in terms of data scale, facilitating a fair feature fusion; (ii) it alleviates gradient explosion or vanishing, which stabilizes the network training; and (iii) the learnable parameter vectors can fine-tune the feature map distributions, actively bridging the gap between static and dynamic deep features. After feature alignment, fusion becomes straightforward. We assume that the feature maps extracted by the two branches are complementary; thus, a simple feature addition operation is used to obtain the fused feature matrix $Z=Z_{s}+Z_{d}$. We present a visualization of the dimensionally-reduced feature Z by t-SNE into a 2D feature space at the right end of Fig. 2(d), where the points of the same organ already begin agglomerating.

### 3.6 Down-stream task module

The DTM of our network (Fig. 2(e)) aims to propagate organ instance labels from $V_{GT}$ to $V_{raw}$ on the crop point cloud, which in essence, assigns category labels of a few nodes (i.e., organ instance labels) to unlabeled nodes in the graph. The fused feature Z is mapped to a node class score matrix $P\in R^{N\times C}$ via equation (11), where C represents the number of node classes—i.e., the number of organ instances.

(11) P=softmax(MLP(Z)).

Finally, for each node (being each row of P), the class with the maximum value is selected as the predicted organ label of that node, which constitutes the output of the entire DBGCN.

In the DTM, we employ the cross-entropy loss function to guide the learning of the network, defined as follows:

(12) $$Loss=-\frac{1}{\left|V_{GT}\right|}\sum_{v=1}^{N}\sum_{c=1}^{C}{\left[P\right]}_{v,c}^{GT}\;\log\;{\left[P\right]}_{v,c}\text{,}$$

where ${\left[P\right]}_{v,c}$ represents the element located at the v-th row and c-th column of matrix P. ${\left[P\right]}_{v,c}^{GT}$ denotes elements of the score matrix corresponding to nodes in $V_{GT}$. The rows corresponding to $V_{GT}$ in P obey one-hot encoding, while the rows corresponding to $V_{raw}$ are zero vectors.

DBGCN efficiently learns with a small amount of supervised information from the labeled point set $V_{GT}$, and the total number of network parameters is only about $3lD^{2}+\left(N+C+5\right)D$. Therefore, the DBGCN network is potentially deployable on non-GPU platforms or edge computing devices.

## 4 Experiments

### 4.1 Dataset preparation

The crop point cloud data used in this study originates from two sources—PlantNet sub-dataset [19,20] and Soybean-MVS sub-dataset [21]. The total dataset has 105 tobacco sample plants, 312 tomato plants, 129 sorghum plants, and 90 soybean plants. All crop point clouds are prepared with point-level organ instance annotations by ourselves, and every sample plant meets the condition of establishing a graph. The per-plant point count of the PlantNet sub-dataset ranges from 5000 to 100,000, which documents the growth of crops over a period of 20 days in different growth environments. In the Soybean-MVS sub-dataset, the original per-plant point count for soybean plant is at the 10,000,000 level, recording the growth of 5 soybean genotypes across two seasons. Given that the original sample plant point clouds are highly dense, the Farthest Point Sampling (FPS) algorithm was employed to down-sample all point clouds in the PlantNet sub-dataset to 2048 points and all crops in Soybean-MVS to 4096 points, aiming to control the scale of adjacency matrices and to improve the computational efficiency. A summary of the dataset is presented in Table 1, covering the number of samples, the point count per-plant, the average number of organ instance per-plant, the maximum and minimum numbers of organ instances per-plant in the sub-dataset, and the maximum and minimum point counts per-organ in the sub-dataset. It is evident that soybean crops in Soybean-MVS have more complex morphologies compared to those in the PlantNet sub-dataset. The complexity of soybean plants justifies why we set the per-plant point count for soybeans twice of the PlantNet.

**Table 1:** Details of our dataset.

Sub-dataset	No. of plants	No. of points per-plant	Mean No. of organs	Max No. of organs	Min No. of organs	Max No. of points of organ	Min No. of points of organ
PlantNet	546	2048	8.51	33	3	1306	1
Soybeans-MVS	90	4096	37.07	95	6	1815	1

**Fig. 3:**
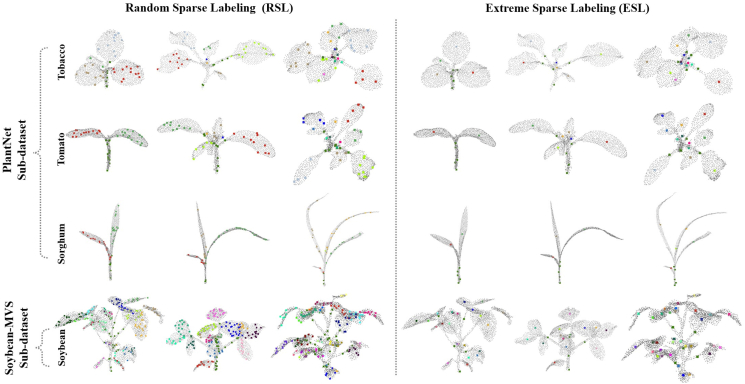
Visualizations of the crop dataset with two different labeling strategies—RSL and ESL. The 1st to 4th rows correspond to the point clouds of tobacco, tomato, sorghum, and soybean species, respectively. We selected three samples from each species to compare the training set $V_{GT}$ generated from two different labeling strategies. The results of RSL are on the left side, those of ESL are on the right. In the displayed point clouds, gray points represent unlabeled test sets, while points of other colors represent training sets with manually added labels. Except from the gray color, different colors correspond to different organ instances.

All points of a crop point cloud form the vertex set V, where the set of points with label information is denoted as $V_{GT}$. The set $V_{GT}$ is also referred to as the training set in subsequent semi-supervised transductive learning. The remaining unlabeled vertex set is $V_{raw}$, acting as the testing set. To simulate how a human annotator may conduct sparse manual labeling for different organs, we designed two sparse label initialization strategies—Random Sparse Labeling (RSL) and Extreme Sparse Labeling (ESL). The distribution visualizations of the two patterns are shown in Fig. 3.

Randomly distributed sparse labels are commonly found in most datasets used for semi-supervised network training and testing, such as the citation datasets Cora and Citeseer [67]. The Random Sparse Labeling strategy is also a frequently used coarse-labeling method among annotators in plant phenotyping. This is because when the geometric overview of the dataset is unclear, annotators tend to first randomly sample points, manually assign labels, and then return the labeled data points back to the dataset. Consequently, the labeled point set $V_{GT}$ spread throughout the entire dataset V. In RSL, for each sample crop in the PlantNet sub-dataset, $\left|V_{GT}\right|$ is set to 40, with labeled points accounting for 1.95% of the total point set. Considering the more complex structure of soybean crops in the Soybean-MVS sub-dataset, the number of labeled points $\left|V_{GT}\right|$ per soybean plant is set to 200, representing 4.88% of the total point set. Additionally, we ensure that each organ of all plants is covered by at least one labeled point. We repeat RSL five times with different random seeds, resulting in five different initial distributions of $V_{GT}$ and $V_{raw}$ for each graph (each plant), denoted as $RS_{i},i\in \left\{1,\ldots ,5\right\}$.

Extreme Sparse Labeling is an efficient labeling strategy for locating crop organ instances in 3D point cloud. ESL is also popular among human annotators as a pre-step when labeling plants. Some annotators tend to first coarsely label each organ with only one point label or one small label area, and then refine all labeled areas to cover all points. This strategy is effective in suppressing mislabeling. We employ different ESL patterns for crop leaves and stem system. For leaves, we only mark the center point of each leaf point set as the sole labeled point, while for the entire stem system of a plant (including the main stem, side branches, and petioles) we randomly label 5 points using FPS. The number of labels formed under ESL strategy is related with the complexity of the crop structure; the more organs, the larger $\left|V_{GT}\right|$ it becomes. It can also be observed from Fig. 3 that the value of $\left|V_{GT}\right|$ under RSL strategy is significantly larger than that of the ESL for the same crop point cloud.

### 4.2 Configuration of hyperparameters and evaluation metrics

In the graph building phase of GRM, we use KNN to establish edges among points (nodes) in the point cloud. The neighborhood size $k_{s}$ for nodes in the PlantNet subset is set to 30 and for the Soybean-MVS subset is 20. The reason for a smaller neighborhood size for Soybean-MVS is that soybean species contain more leaves than other crops, and a larger neighborhood might introduce category noise from surrounding organs. Subsequently, during the input reshaping phase, to compute the similarity matrix S for input nodes using equation (1), the Gaussian kernel parameter kernel is set according to the recommendation in Ref. [68]:

(13) $$kernel=2\sum_{v=1}^{N}\sum_{u=1}^{k_{s}}d_{vu}/Nk_{s}\text{.}$$

Concatenating S after the crop point cloud coordinate matrix P yields the input feature X, and the subsequent network can fully utilize the node 3D coordinates as well as the embedded local neighborhood information from X.

In DBGCN, all the feature dimensions in hidden layers are fixed to D. Considering the differences between sub-datasets, D is set to 64 in the PlantNet subset and 128 in the Soybean-MVS subset. The remaining hyperparameters in SGCB follow the default configuration of GCNII. For convenience, the dynamic neighborhood size $k_{d}$ of nodes in DGCB is consistent with $k_{s}$. Additionally, the number of graph convolution layers is the same in both branches. Two types of scenarios under l=2 and l=4, are compared in experiments. The network's learning rate is set to $5\cdot e^{-4}$.

To validate the effectiveness of the proposed DBGCN, we compare it with popular graph transductive models including GCN [16], GCNII [17], GAT [41], Grand [18], and JKnet [44], the inductive GraphSage [40] model, as well as inductive deep networks PlantNet [14] and PSegNet [15]. Transductive models train and test on a single graph (a sample plant) without inheriting network parameters learned from the previous graph. The inductive GraphSage model has two types of learning strategies. The first one is named as “GraphSage-1”, which trains and tests on each single graph of plant using the data labeling strategies in Section 4.1. The GraphSage-1 uses the same reshaped input feature X as our network; however, the training set and testing sets are completely separated, with no adjacency connections between nodes from the two sets, which is different from our DBGCN. The second is named as “GraphSage-2”, which also trains and tests on each single graph of plant, but divides the graph into the training set and the testing set with a 2:1 ratio based on node count, and the input uses only the coordinate matrix P. The inductive deep segmentation networks PlantNet and PSegNet divide all point clouds into a training set group and a testing group with a 2:1 ratio in the number of point clouds, and perform typical inductive deep learning with pure coordinates P as input feature. Considering that GAT is good at learning the similarity across neighboring nodes directly from 3D coordinates, we additionally implemented a GAT version—GAT (P) that uses only pure coordinate feature P as input. Furthermore, due to the extremely sparse supervision information and the shallow architecture of GCNs, Dropout operations may lead to the loss of key supervision signals. Therefore, the Dropout layers of all networks were disabled in the experiments to ensure the integrity of sparse supervision signals.

All experiments in this section were conducted on a workstation equipped with a 16-core CPU, 128 GB memory, and 4 paralleled NVIDIA GeForce RTX 2080Ti GPUs. The system is operated by Ubuntu 20.04 OS, and the deep network is implemented with Pytorch 1.7.1. All training details of the deep networks used recommended parameters from their original papers, respectively. The total number of epochs for the DBGCN model is set to 200, and the epoch achieving the best evaluation metrics during training is selected as the final model.

The organ inference task based on sparse annotation in crop point cloud is essentially a node classification task on graph data; thereby, the node classification accuracy Acc calculated by equation (14) can be used as an evaluation metric:

(14) $$Acc=\frac{TP}{TP+FP}\times 100\%\text{,}$$

in which TP represents the number of nodes correctly classified by the model, and FP represents the number of nodes incorrectly classified as the current organ by the model. Acc is actually the proportion of correctly classified points of an organ to the total points of that organ inferred by the network, represented in a percentage. A closer Acc value to 1.0 stands for a better performance of the model.

### 4.3 Quantitative results of organ instance inference

This subsection shows the quantitative comparison between our DBGCN and eight other models/networks on the dataset. The quantitative results of organ label propagation on the testing set $V_{raw}$ of two sub-datasets under RSL are shown in Table 2, in which the two parameters in the parentheses after the network name are the number of network layers and the input form. For example, GAT (l=2, X) means that the GAT network is set to 2 layers and uses X as the input feature. All transductive networks independently conducted the total experiment under RSL for five times, and “mAcc” represents the mean accuracy of node classification across the five experiments. Under RSL, the two-layer DBGCN (l=2) achieved a mAcc of 92.64% on the PlantNet sub-dataset, outperforming the second best GCNII by 1.16%; while the four-layer DBGCN (l=4) reached 93% mAcc, outperforming the second best GCNII (l=4) by 0.99%. On the Soybean-MVS sub-dataset with RSL, the two-layer DBGCN (l=2) achieved mAcc at 90.49%, outperforming the second best GCN by 2.93%; while the four-layer DBGCN (l=4) achieved mAcc at 91.05%, outperforming the second best GCNII by 2.03%. The performance of inductive GraphSage-based methods and inductive deep networks PlantNet and PSegNet are not as good as the transductive counterparts, especially on the soybean crops. We did not list the results of l=4 for several networks in Table 2 because their results were even worse than their l=2 versions.

**Table 2:** Quantitative comparison results of organ label propagation under RSL strategy. The best result is in bold, and the second best is underlined.

Learning paradigm	Methods	PlantNet sub-dataset	Soybean-MVS sub-dataset
		mAcc (%)	mAcc (%)
Transductive	GCN (l=2, X)	90.46	87.56
	GCNII (l=2, X)	91.48	83.30
	GAT (l=2, X)	90.71	84.24
	GAT (l=2, P)	87.22	79.34
	Grand (l=2, X)	90.47	81.41
	JKnet (l=2, X)	91.26	88.20
	Our DBGCN (l=2, X)	92.64	90.49
	GCNII (l=4, X)	92.01	89.02
	JKnet (l=4, X)	91.43	88.55
	Our DBGCN (l=4, X)	**93.00**	**91.05**
Inductive	GraphSage-1(l=2, X)	87.52	62.75
	GraphSage-2 (l=2, P)	90.37	74.66
	PlantNet (P)	88.47	64.95
	PSegNet (P)	88.56	65.77

The quantitative experimental results under ESL are shown in Table 3. Due to the highly limited labeled data $\left|V_{GT}\right|$, inductive methods like GraphSage-2 are unable to operate. Our DBGCN remains the best across all methods compared. In the PlantNet sub-dataset under ESL, the mAcc of two-layer DBGCN is 88.60%, which is evidently superior to the second best GAT by 6.8%. The mAcc of our four-layer DBGCN is 89.01%, outperforming the second best GCNII by 6.5%. In the Soybean-MVS sub-dataset under ESL, the mAcc of two-layer DBGCN is 82.96%, outperforming the second best GCN by 7.18%; the four-layer DBGCN achieves mAcc at 84.49%, outperforming the second best GCNII by 9.6%. Under ESL strategy, the scarcity of the supervision signals can cause the network to overfit on the training set $V_{GT}$, which significantly reduces the performance of GAT(l=2, P) that aggregates local neighborhoods using multi-head attention. It is noteworthy that using X on the GAT network as input feature is superior to using 3D coordinates P; and this indirectly proves the superiority of our input feature reshaping process in GRM in subsection 3.3. The Grand network employs a perturbation enhancement mechanism, which may lose critical graph structure information on complex crop structures. The inductive GraphSage-1 performs the worst under ESL because it only learns from very sparse node features of the training set that appears as isolated “islands” from the perspective of the entire crop graph. It is difficult to correctly propagate information to other point cloud regions outside these “islands”.

**Table 3:** Quantitative comparison results of organ label propagation under ESL strategy. The best result is in bold, and the second best is underlined.

Methods	PlantNet sub-dataset	Soybean-MVS sub-dataset
	mAcc (%)	mAcc (%)
GCN (l=2, X)	81.72	75.78
GCNII (l=2, X)	81.74	69.26
GAT (l=2, X)	81.80	74.42
GAT (l=2, P)	73.41	63.45
Grand (l=2, X)	80.41	68.65
JKnet (l=2, X)	78.83	71.02
GraphSage-1(l=2, X)	74.81	56.02
Our DBGCN (l=2, X)	88.60	82.96

GCNII (l=4, X)	82.51	74.89
JKnet (l=4, X)	79.22	72.81
Our DBGCN (l=4, X)	**89.01**	**84.49**

As stated above, our DBGCN performs best in all quantitative experiments across all models compared.

### 4.4 Qualitative results of organ instance inference

**Fig. 4:**
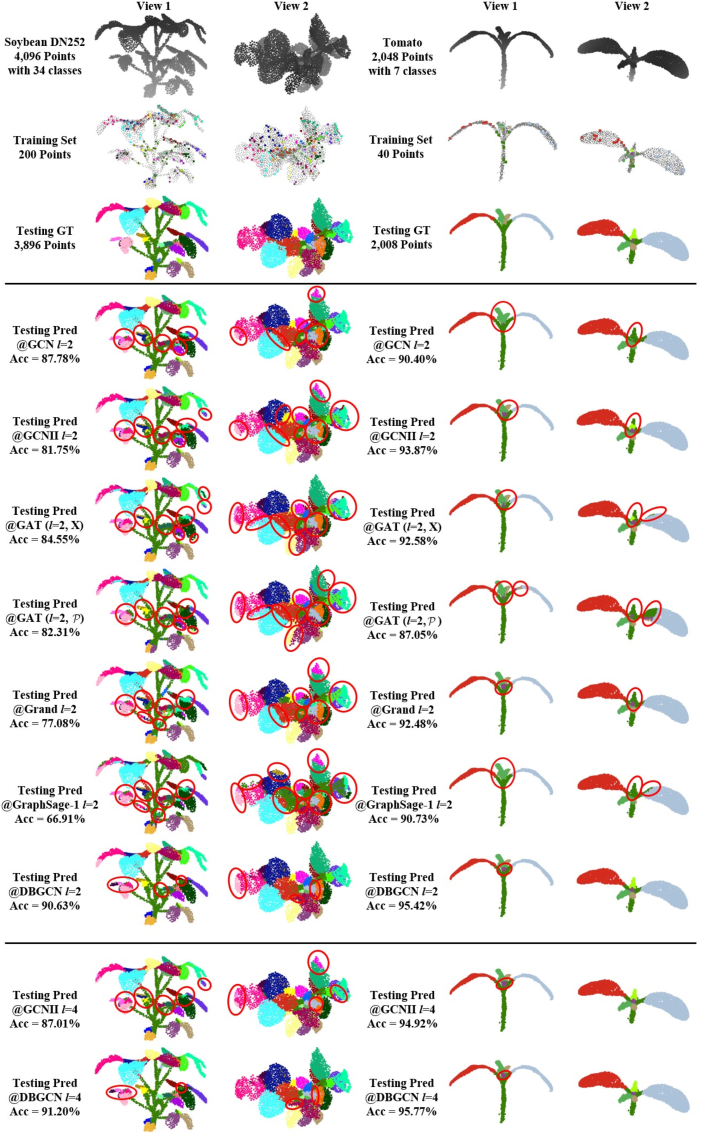
A qualitative comparison of organ label propagation results under RSL strategy on two sample plants. View1 and view2 represent two different viewing perspectives. The Training set is $V_{GT}$, while the Testing GT represents the visualization of the true label values for the testing set $V_{raw}$. Testing Pred is the visualization of the predicted node labels by the network, and red circles indicate incorrect areas of prediction. Acc means node classification accuracy for one point cloud.

**Fig. 5:**
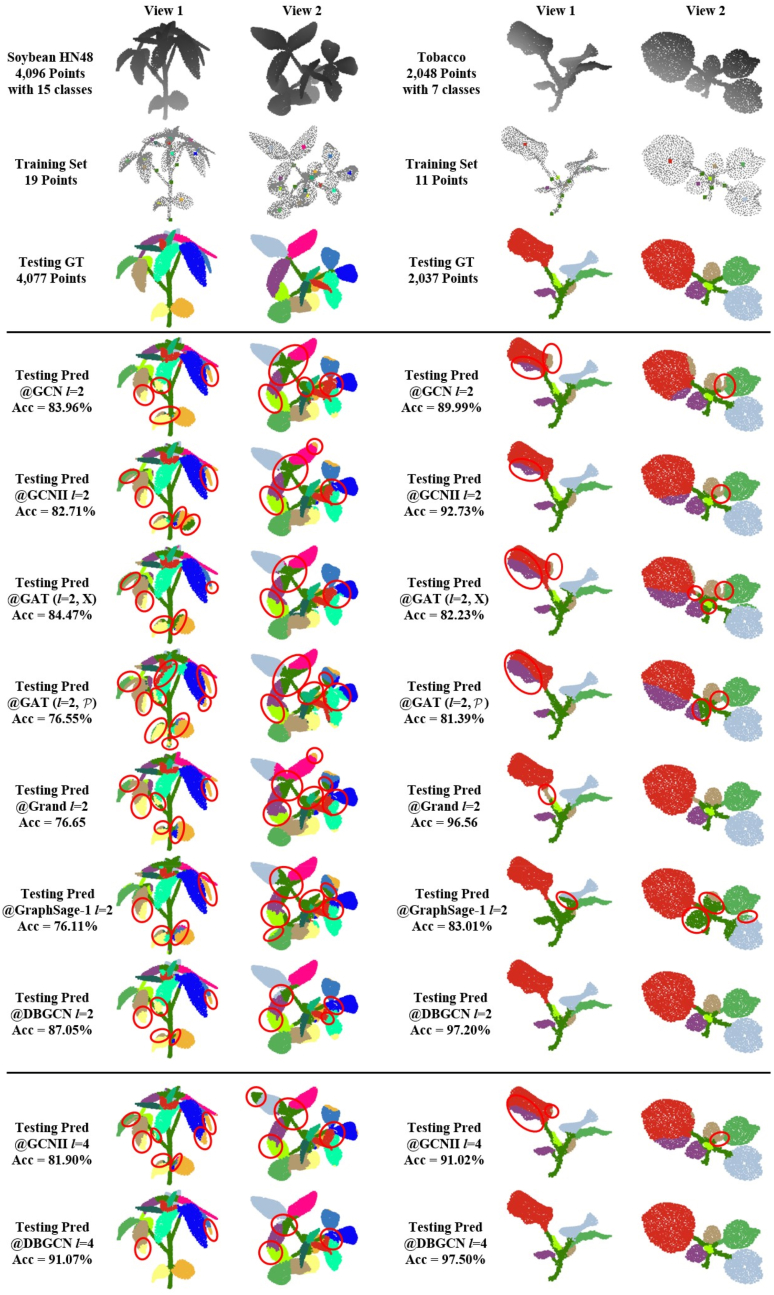
A qualitative comparison of organ label propagation results under ESL strategy on two sample plants. View1 and view2 represent two different viewing perspectives. The Training set is $V_{GT}$, while the Testing GT represents visualization of the true label values for the testing set $V_{raw}$. Testing Pred is the visualization of the predicted node labels by the network, and red solid circles indicate incorrect areas by prediction. Acc means node classification accuracy for one point cloud.

To visually demonstrate the label propagations of multiple transductive methods under two sparse labeling strategies, this subsection qualitatively presents results using several representative crops. Fig. 4 shows the qualitative comparison of label propagation results for all compared models under RSL on a DN252 sample (containing 34 organs) from Soybean-MVS sub-dataset and a tomato sample (7 organs) from PlantNet sub-dataset. The qualitative comparison of label propagation results for all models under the ESL strategy on a HN48 sample (containing 15 organs) from Soybean-MVS sub-dataset and a tobacco sample (7 organs) from PlantNet sub-dataset are shown in Fig. 5.

As shown in Fig. 4, the soybean crop has a complex structure with numerous branches and serious leaf overlapping, making it difficult for correct organ label inference. Several models even output obvious classification errors at the tips of large leaves. For tomato crops, segmenting the small buds at the top of the main stem is challenging, and most compared methods smoothed the top details as a single leaf. Our DBGCN can adapt to different structures of crop species, demonstrating the best label propagation visualization results. The DBGCN results for the soybean DN252 sample have the fewest incorrect areas, with only six minor errors at two-layer network depth and it further reduces to four errors at four-layer network depth. On the tomato sample, the accuracy of DBGCN at both depths exceed 95%, approaching the human annotation quality. The minor errors only occurred at the very tip of the bud.

Among the results under ESL shown in Fig. 5, the two-layer DBGCN achieves an accuracy of 87.05% for the soybean HN48 sample, and the four-layer DBGCN further improves the accuracy to 91.07%. The area of error regions of DBGCN are the smallest across all the compared methods under ESL. In the results of other models, it can be observed that soybean leaves are prone to fragmentation issue—a large leaf is usually broken into pieces, especially around leaf edges. For the tobacco sample plant having merely 11 labeled points as the training set, both versions of our DBGCN exceed 97%, which are very close to manual annotations.

In summary, as a result of the dual-branch architecture combining dynamic and static graph convolution calculations, DBGCN is capable of propagating crop organ labels from very extremely sparse annotation, and also correctly determining the classification boundaries among different organs. Additionally, DBGCN is competent for capturing local structural details (e.g., leaf edges and boundaries among leaves) while ensuring global structural perception.

### 4.5 Quality tests of the data labeled by DBGCN

**Fig. 6:**
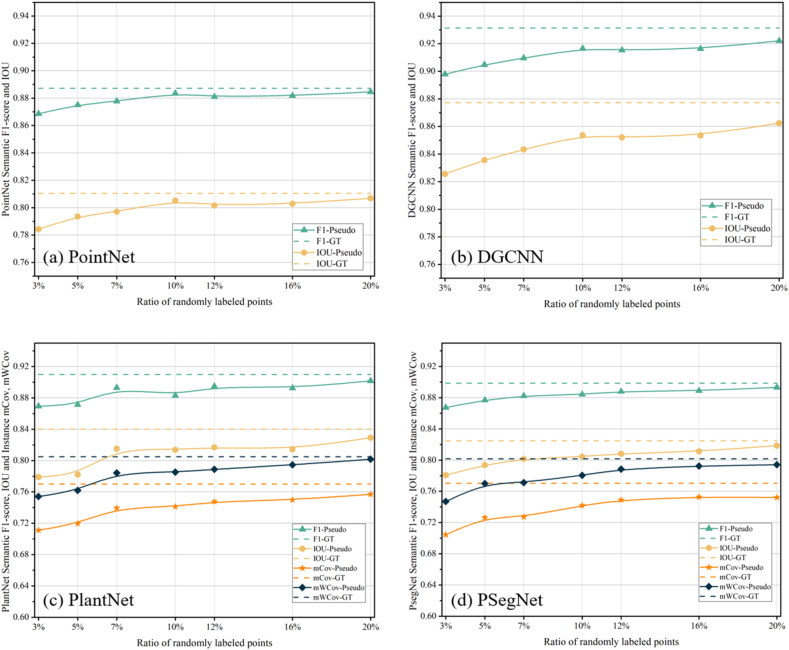
Quantitative performance comparison of inductive deep networks based on fully-labeled dataset generated at different ratio values of $\left|V_{GT}\right|/\left|V\right|$ on DBGCN. (a) is the results of the PointNet organ semantic segmentation network, (b) is the results of the DGCNN organ semantic segmentation network, (c) is the results of PlantNet dual-functional organ segmentation network, and (d) is the results of PSegNet dual-functional organ segmentation network. The curves in the figure are fitted based on the data points of the evaluation metrics, and the dotted horizontal lines represent the results of the network trained by pure GT data.

To further validate the quality of the full coverage of point-level labels output by DBGCN, we used the fully-labeled dataset by DBGCN to train inductive deep networks for inductive organ segmentation, and compared the performance against the corresponding networks trained by GT data purely labeled by human. In addition, in order to find the best ratio of annotation $\left|V_{GT}\right|/\left|V\right|$ in RSL for DBGCN, we conducted tests by applying different ratio values on DBGCN to generate different fully-labeled datasets, and these datasets were lately used for inductive deep segmentation. The quality test process involved the following steps. First, we applied a four-layer DBGCN to the PlantNet sub-dataset under RSL with a given labeling ratio $\left|V_{GT}\right|/\left|V\right|$, and performed label propagation to obtain a fully-labeled plant dataset that is called “Pseudo” dataset. Second, the “Pseudo” datasets generated at different $\left|V_{GT}\right|/\left|V\right|$ ratios, along with the GT dataset, were separately fed into a inductive deep organ segmentation network (our network choice includes PointNet [39], DGCNN [40], PlantNet [14], and PSegNet [15]) for training. Finally, quantitative evaluations were performed on the testing results from those trained models. For the two networks—PointNet and DGCNN that can only conduct organ semantic segmentation, the evaluation metrics are F1-score and IOU [14,15]. For PlantNet and PSegNet—two networks supporting both organ semantic and instance segmentation, additional metrics including the mean coverage (mCov) and the mean weighted coverage (mWCov) [14,15] were used. As shown in Fig. 6, the performances of all four deep networks trained with “Pseudo” datasets improve with the increasing $\left|V_{GT}\right|/\left|V\right|$ ratio, and the four network performances gradually approaches results obtained using GT data. When the ratio is between 5% and 10%, the quantitative metrics from “Pseudo” datasets are already comparable to those from GT. When the ratio of $\left|V_{GT}\right|/\left|V\right|$ reaches 20%, the F1-score and IOU of PointNet trained by GT data outperforms the “Pseudo” data counterpart by only 0.26% and 0.36%, respectively; the F1-score and IOU of DGCNN trained by GT data outperforms the “Pseudo” data counterpart by only 0.93% and 1.48%, respectively. At the ratio of 20%, the mCov and mWCov of PlantNet for GT data outperforms the “Pseudo” data counterpart by 1.29% and 0.33%, respectively; and the mCov and mWCov of PSegNet for GT data outperforms the “Pseudo” data counterpart by only 1.83% and 0.76%, respectively. In conclusion, DBGCN can generate high-quality full coverage of organ labels on plants based on only sparse manual annotation, which significantly reduces the human-guided data annotation workload in inductive deep learning for point clouds.

## 5 Discussion

### 5.1 Parameter tuning

#### 5.1.1 Influence from the shape of input

To validate the advantage of the GRM proposed in subsection 3.3, we conducted an experiment comparing the performances of several versions of DBGCN using different input shapes on the Soybean-MVS sub-dataset. The experimental setup followed the same single-graph (single-plant) transductive learning paradigm, employing a four-layer DBGCN architecture averaged on five independent RSL tests. Each point cloud has 200 randomly selected nodes as the training set $V_{GT}$, and the remaining nodes serving as the test set $V_{raw}$. Four different input features were compared in the label propagation experiment, with quantitative results presented in Table 4. In the results, P represents the 3D coordinate features of the point cloud; S denotes the row-normalized Gaussian kernel spatial similarity matrix S; P⊕norm(A) represents the concatenation of P and the row-normalized adjacency matrix; and the last P⊕S denotes the concatenation of P and S, which is also the proposed input shape in this study. As shown in Table 4, the P⊕S version achieves the highest mAcc of 91.21% by DBGCN, outcompeting the pure coordinate feature P version by 16.85%; this proves the importance of introducing the node similarity feature. Using S alone as input still outperforms P by 16.04%, indicating the strong ability of similarity matrix in modeling local geometric relationships in node classification. The accuracy of P⊕norm(A) is slightly lowers than P⊕S but still superior to the version that only contains S, showing the usefulness of low level features—the pure 3D coordinates. The advantage of the proposed P⊕S input shape indicates that both the low-level position information and the smoothed neighborhood information are useful in graph learning.

**Table 4:** Quantitative comparison of four input shapes for our DBGCN on the Soybean-MVS sub-dataset. Hereby, RS_i_ denotes the i-th independent experiment with RSL data initialized with an unique random seed. The last column shows the average of five independent experiments. The best value is in bold, and the second best is underlined.

Feature input	RS_1_ Acc	RS_2_ Acc	RS_3_ Acc	RS_4_ Acc	RS_5_ Acc	mAcc
P	77.21	77.30	77.23	77.32	77.15	77.24
S	89.55	89.77	89.76	89.84	89.51	89.69
P⊕norm(A)	90.78	90.94	90.64	90.82	90.90	90.82
X=P⊕S	**90.92**	**91.12**	**90.86**	**91.20**	**91.06**	**91.03**

#### 5.1.2 Tuning of K-neighbors, the feature dimensionality, and the smoothness kernel

**Fig. 7:**
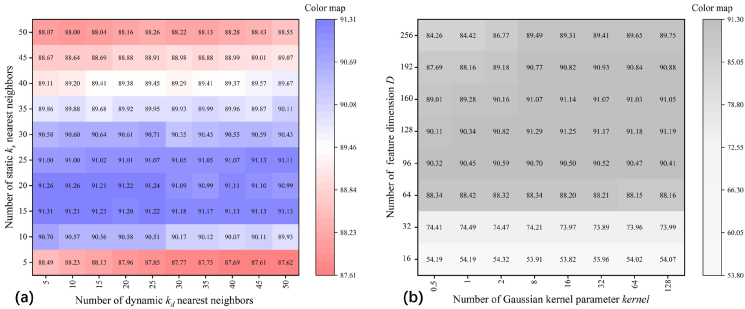
Tuning experiments for multiple parameters including $k_{s}$, $k_{d}$, D, and kernel. (a) shows the 2D tuning test for $k_{s}$ and $k_{d}$ in a colored matrix (in which more bluish means better), and (b) shows the 2D tuning test for D and kernel in a grayscale matrix (in which darker means better). All values in the two matrices represent the Acc (%) from the DBGCN network with the given parameters. Acc means node classification accuracy for one point cloud.

In this paragraph we first discuss the tuning of “K-neighbors” in KNN calculations. The parameter $k_{s}$ in SGCB and parameter $k_{d}$ in DGCB from the Dual-branch Graph Convolutional Module can influence their respective convolution calculations. We performed separate tuning experiments on a 4-layer DBGCN architecture using Soybean-MVS sub-dataset under RSL strategy. The neighborhood parameter values started from 5 and increased by a step of 5 until 50. The results are shown in the colored matrix in Fig. 7(a), where the elements represent Acc values of the network organ inference based on different parameter settings, respectively. It can be observed that when adjusting $k_{d}$ under a fixed $k_{s}$ (horizontal direction), the performance of DBGCN exhibits a low variance, indicating the robustness of DGCB to the choice of $k_{d}$. And this also indirectly suggests that the dynamic-feature-space graph convolution has strong adaptability to neighborhood noise. When adjusting $k_{s}$ under a fixed $k_{d}$ (vertical direction), the distribution of Acc numbers in each column takes a Gaussian shape; i.e., higher values in the middle and lower values on both sides. When $k_{s}<10$, the network performance declines, and this may primarily due to that the small receptive field of static graph convolution preventing the network from perceiving complete global structural information. When $k_{s}>30$, the network performance also declines, which possibly is because that an excessively large receptive field can lead to feature over-smoothing in graph convolutions. Additionally, larger neighborhoods are generally disadvantageous for the network to perform fine-grained node label classification at organ boundaries. For convenience, $k_{s}$ and $k_{d}$ can be set to a same value ranging from 10 to 30, and this strategy helps the network to achieve optimal performance.

Now we begin the tuning of the feature dimensionality D and the smoothness kernel parameter kernel. We still performed separate tuning experiments on a 4-layer DBGCN architecture using Soybean-MVS sub-dataset under RSL strategy. The two parameters are also tuned collaboratively in a 2D grayscale matrix shown in Fig. 7(b). When carrying out the tuning, both $k_{s}$ and $k_{d}$ were fixed to 30. The dimensionality parameter D was evaluated at eight different numbers—16, 32, 64, 96, 128, 160, 192, and 256. The kernel parameter was also evaluated at eight levels—0.5, 1, 2, 8, 16, 32, 64, 128. It can be first observed from Fig. 7(b) that the accuracy is quite robust to the variations of kernel, indicating the choice of this parameter is flexible in practice. However, when it comes to parameter D, the situation is very different. With the increasing of the dimensionality, the performance of DBGCN quickly goes up and then falls down, peaking at around D=128. Therefore, we suggest that the dimensionality parameter to be set around 128 for optimal performance.

#### 5.1.3 The influence of $\left|V_{GT}\right|$ on the performance of DBGCN

**Fig. 8:**
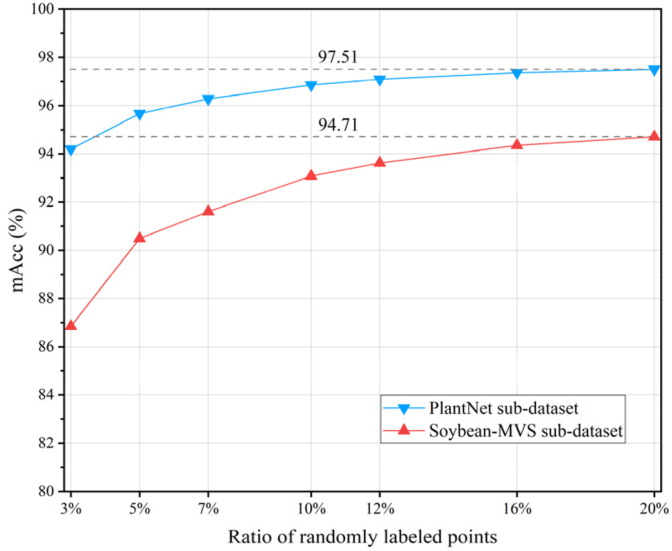
The influence of annotation scale $\left|V_{GT}\right|$ on DBGCN performance on two sub-datasets. The horizontal axis represents the annotation ratio $\left|V_{GT}\right|/\left|V\right|$, and the vertical axis represents mAcc by DBGCN. As all plant samples in each sub-dataset have the same number of nodes |V|, the ratio of $\left|V_{GT}\right|/\left|V\right|$ functions the same as $\left|V_{GT}\right|$.

In the label propagation task, the size of the training set has a direct impact on transductive models. To observe the impact of randomly annotated data scale on DBGCN, training sets with different annotation scale $\left|V_{GT}\right|$ were generated under varying labeling ratios, and each quantitative result was averaged on five independent tests with a two-layer DBGCN. The curves for organ label propagation under different $\left|V_{GT}\right|$ with two sub-datasets are shown in Fig. 8, where mAcc exhibits a three-stage increasing trend as the annotation scale $\left|V_{GT}\right|$ increases. Stage 1 is the rapid increase period (annotation ratio between 3.0% and 5.0%). In this stage, mAcc increases almost linearly (with a high slope) with the increase of annotation scale. In stage 1, the accuracy on PlantNet sub-dataset improves by 2.66%, while Soybean-MVS sub-dataset improves by 6.24%, indicating that increasing $\left|V_{GT}\right|$ in this range effectively enhances model performance. Stage 2 (annotation ratio between 5.0% and 12.0%) is a slow growth period. As $\left|V_{GT}\right|$ increases, the mAcc increases almost linearly in a slow speed. Within this stage, mAcc increases by approximately 1.4% for PlantNet sub-dataset and about 3.0% for Soybean-MVS sub-dataset. Stage 3 is the saturation period (labeling ratio above 12.0%). The growth of mAcc tends to saturate when $\left|V_{GT}\right|$ continues to increase. For PlantNet sub-dataset, mAcc reaches its highest value at 97.51% when the annotation rate reaches 20%, while mAcc reaches 94.71% for the Soybean-MVS sub-dataset. The curves in Fig. 8 indicates that the main capability of model is already activated when $\left|V_{GT}\right|$ accounts for 5% to 10% of the total nodes. Notably, compared to the 5% annotation ratio, though the 20% ratio can further increase the DBGCN performance on the two sub-datasets, the manual workload of labeling at also increases to four times of the 5% ratio. This explains why the default RSL used annotation ratio around 5%, which achieves a good trade-off between labeling cost and network performance.

### 5.2 Ablation study

We designed an ablation study in this subsection to verify the effectiveness of the main modules in DBGCN, including removal of the DGCB branch, removal of the SGCB branch, replacing the static-feature-space convolutions in SGCB with a standard GCN, and removal of the feature alignment step in the FM. Including the standard (complete) DBGCN network, we will compare a total of 5 model versions quantitatively. The ablation study uses a 4-layer DBGCN as baseline and conducts 5 repeated RSL tests on a Soybean-MVS subset. The quantitative experimental results are shown in Table 5. Among them, “w/o DGCB” means removal of the 4-layer DGCB from the baseline; “w/o SGCB” represents removal of the 4-layer SGCB branch from the baseline; “w/o Alignment (fusion)” refers to removal of the feature alignment step in the FM, and the features from the two branches are directly added and processed during feature fusion; “w GCN” denotes that the 4-layer SGCB branch is replaced by a standard 4-layer GCN; “Full” is the complete DBGCN network as baseline. It can be seen that the complete network achieves the best performance, and removing any module will result in a decline in performance. “w/o SGCB” performs the worst, possibly because using only dynamic graph convolutions tends to overlook the global graph structure.

**Table 5:** Ablation study on DBGCN.

Model Ver	RS_1_ Acc	RS_2_ Acc	RS_3_ Acc	RS_4_ Acc	RS_5_ Acc	mAcc (%)
w/o DGCB	90.21	90.45	90.27	90.45	90.36	90.35
w/o SGCB	69.99	70.15	69.66	70.06	69.81	69.93
w/o Alignment (fusion)	86.29	86.64	86.57	86.80	86.61	86.58
w GCN	89.05	89.14	89.20	89.34	89.20	89.19
Full	**90.98**	**91.22**	**90.89**	**91.12**	**91.04**	**91.05**

### 5.3 Data generalization ability

This subsection discusses the generalization ability of DBGCN on datasets outside the plant phenotyping field. We evaluated DBGCN on a large-scale high-precision LiDAR outdoor point cloud dataset Paris-Lille-3D [69]. The proposed DBGCN achieves satisfactory semi-supervised node inference on high-precision Paris-Lille-3D dataset, demonstrating its good generalization capability on non-agricultural 3D data. The detailed experimental results on this dataset can be found in Appendix 7.2.

## 6 Conclusion

To address the annotation challenge that almost all mainstream inductive learning networks face in the crop organ instance segmentation task, this paper proposes a novel Dual-branch Graph Convolutional Network (DBGCN). This network adopts a transductive learning paradigm, enabling direct organ instance inference (segmentation) on each single plant point cloud with highly sparse labels. Experimental results on crop point clouds of multiple species show that DBGCN achieves satisfactory organ instance segmentation accuracy, outperforming several popular transductive graph networks and even mainstream inductive deep learning architectures.

Despite the fast inference speed (less than 1 min on a crop point cloud) and the effective organ instance segmentation performance, currently our DBGCN only accepts a graph input having less than 50,000 nodes (N⩽50000). The computation load is sensitive to the scales of the input X, the similarity matrix $S^{N\times N}$, and the adjacency matrix $A^{N\times N}$. In the future, we are interested in designing decomposing techniques to divide the direct feature calculation into easily calculable sub-tasks, allowing DBGCN's application to even bigger graphs. We will also continue to improve the internal modules of DBGCN by adding transformer-like components. Additionally, we will optimize the way we implement graph convolutions within the model to enable DBGCN to run on highly complicated and large-scale point clouds.

## CRediT authorship contribution statement

Dawei Li: Conceptualization, Visualization, Validation, Methodology, Investigation, Formal analysis, Funding acquisition, Writing – original draft, Writing – review & editing. Zhaoyi Zhou: Writing – original draft, Validation, Methodology, Investigation, Data curation. Si Yang: Funding acquisition, Data curation. Weiliang Wen: Writing – review & editing, Writing – original draft, Visualization, Formal analysis.

## Data availability

Our data and code are available at: https://github.com/chinazhouzhaoyi/DBGCN/tree/master/.

## Declaration of competing interest

The author is an Editorial Board Member/Editor-in-Chief/Associate Editor/Guest Editor for this journal and was not involved in the editorial review or the decision to publish this article.

The authors declare the following financial interests/personal relationships which may be considered as potential competing interests: Given his roles as Associate Editor, Weiliang Wen had no involvement in the peer review of this article and had no access to information regarding its peer review. Full responsibility for the editorial process for this article was delegated to another journal editor.

## Figures and Tables

**Fig. A1:**
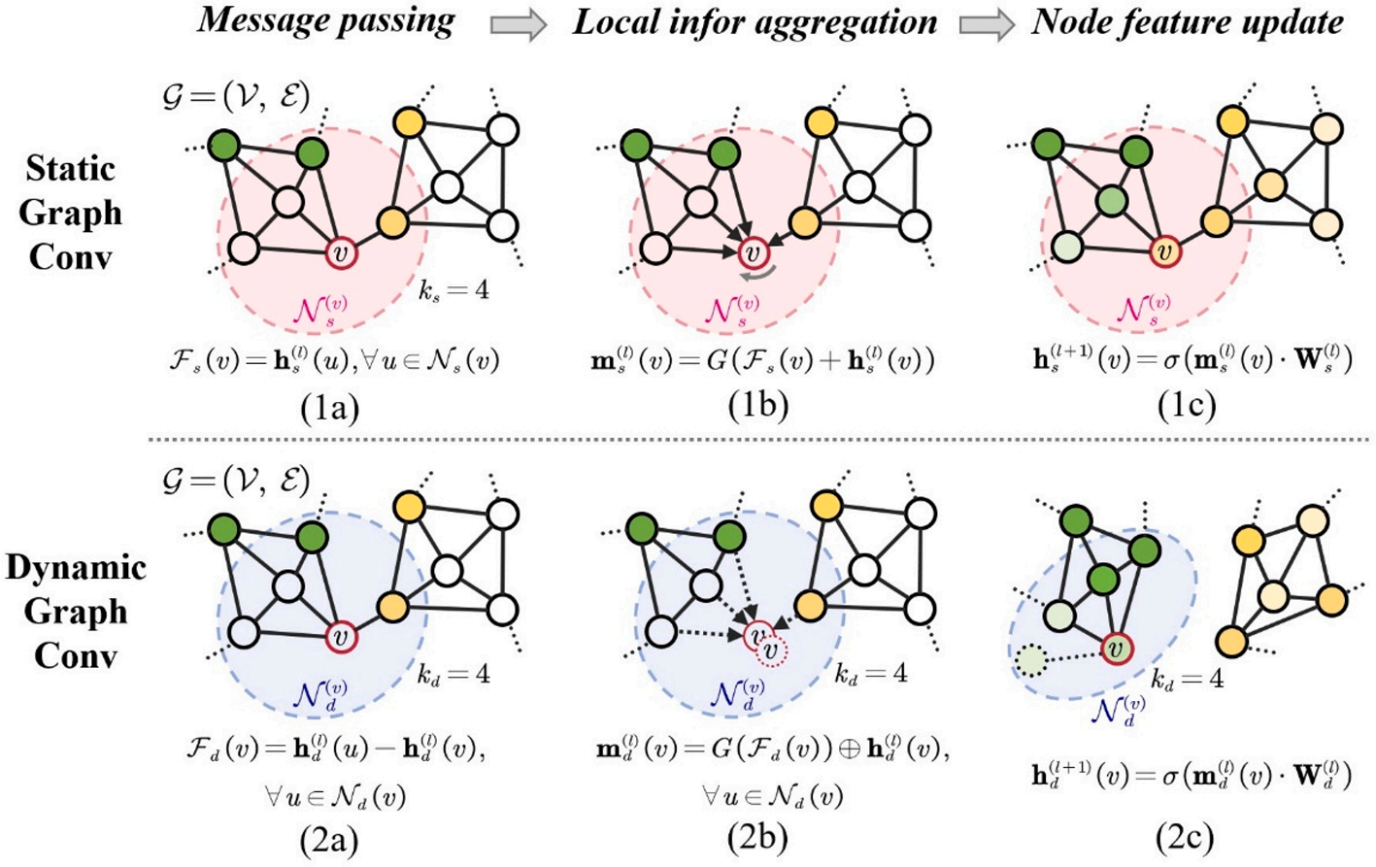
An illustration of the static and dynamic graph convolutions on the same graph G=(V,E). (1a) illustrates the message passing phase of static graph convolution, (1b) depicts the local information aggregation phase of static graph convolution, and (1c) shows the node feature update of static graph convolution. (2a), (2b), and (2c) show the message passing, local information aggregation, and node feature update of dynamic graph convolution, respectively. For static graph convolution, the $N_{s}\left(v\right)$ remains unchanged during computation. For dynamic graph convolution, the $N_{d}\left(v\right)$ differs in every single layer.

**Fig. A2:**
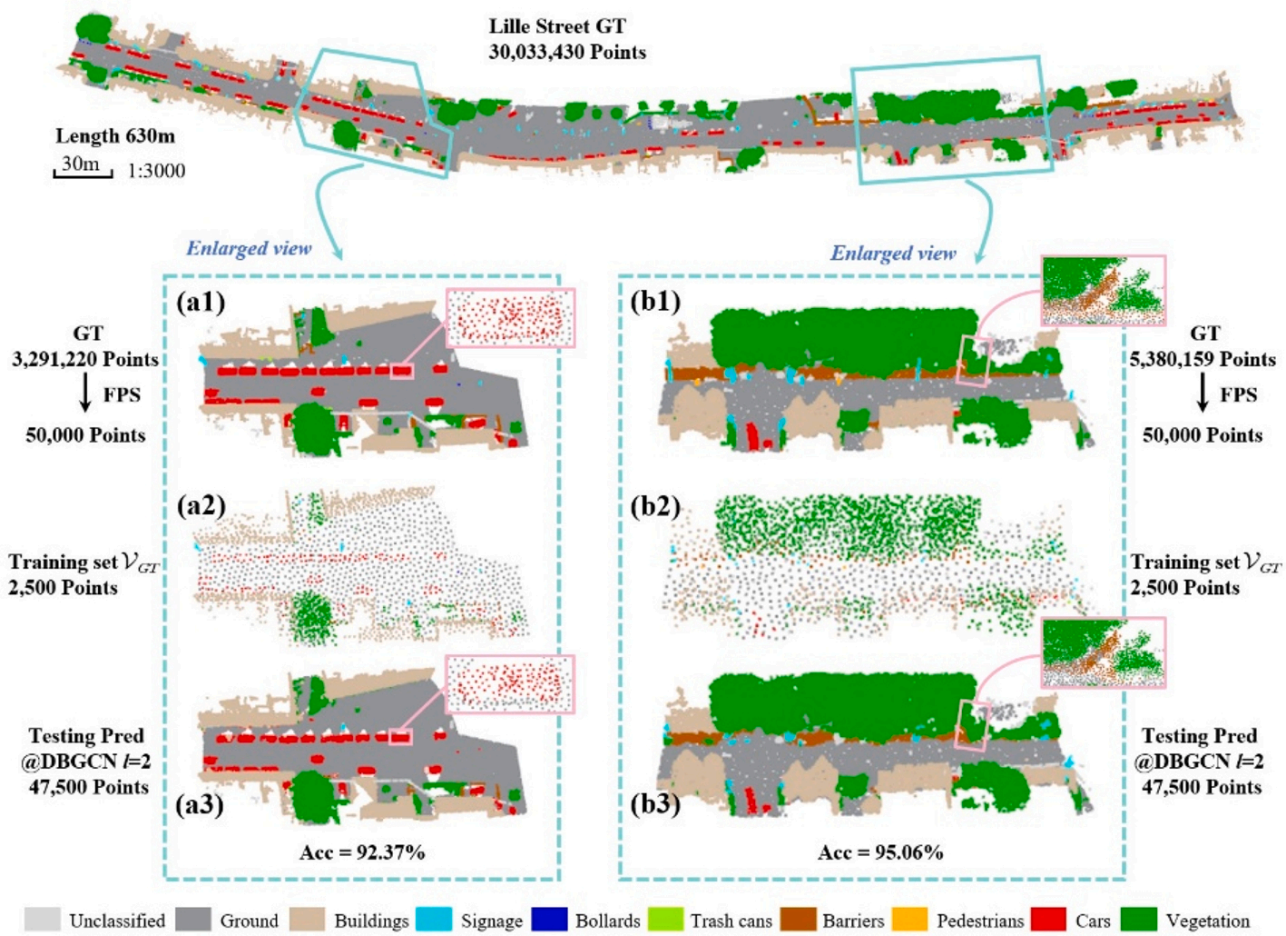
The DBGCN label propagation experiment on the Lille1 street scene. The annotated semantic GT of the street scene point cloud contains 10 types of objects, corresponding to the 10 color indices at the bottom of the figure. The two blue bounding boxes represent two extracted slices, containing 3,291,220 and 5,380,159 labeled points, respectively. (a1) and (b1) show the two slices, each containing 50,000 points (down-sampled by FPS) as point set V. (a2) and (b2) represent two 2500-point training sets formed by RSL from (a1) and (b1), respectively. (a3) and (b3) visualize the label propagation results by DBGCN on the two slices. The Acc for (a3) and (b3) are 92.37% and 95.06%, respectively. Additionally, we use pink boxes to help comparing the details of the scenes. The DBGCN results are similar to GT.

## Appendix

### 1 The comparison of the Static-feature-space graph convolution and the Dynamic-feature-space graph convolution

In the DBGCM of this network, static-feature-space graph convolution and dynamic-feature-space graph convolution are computed separately. The calculation processes of static and dynamic convolutions are visualized in Fig. A1. Both types of convolution calculations can be generally divided into three steps—message passing, local information aggregation, and node feature update. Static graph convolution is performed on the fixed neighborhood structure of the initial feature graph, while dynamic graph convolution is performed within the node neighborhood in the current feature space that is constantly changing when the calculation goes deeper.

First, we focus on the calculation process of each layer in static graph convolution. DBGCN uses equation (4) to compute the static graph convolution at each layer, which is similar to the feature update process in GCNII. If equation (4) is simplified to a basic GCN, the static graph convolution takes the following form in equation (A-1):

(A-1) $$H_{s}^{\left(l+1\right)}\approx \sigma \left(\tilde{A}\cdot H_{s}^{\left(l\right)}\cdot W_{s}^{\left(l\right)}\right)\text{.}$$

We now attempt to unfold the full feature matrix calculation of (A-1) to each individual node feature. Without loss of generality, we discuss the target node v at the l-th layer, whose KNN neighborhood in the initial input feature layer $H_{s}^{\left(1\right)}$ is denoted as $N_{s}\left(v\right)$ with a fixed neighborhood parameter $k_{s}$. It is not difficult to see that $N_{s}\left(v\right)$ corresponds to the v-th row (and also the v-th column) of the full graph adjacency matrix A. The static graph convolution in (A-1) essentially first uses the feature vectors of the target node neighborhood directly as the neighborhood feature transformation function $F_{s}\left(\cdot \right)$ for message passing:

(A-2) $$F_{s}\left(v\right)=h_{s}^{\left(l\right)}\left(u\right)\forall u\in N_{s}\left(v\right)\text{.}$$

In the subsequent local information aggregation step, the self-loop of the target node v is added to the neighborhood features, and the arithmetic mean function G(·) is used to perform feature aggregation. At this point, we obtain the static local neighborhood feature $m_{s}^{\left(l\right)}\left(v\right)$ of the target node v at the l-th layer as:

(A-3) $$m_{s}^{\left(l\right)}\left(v\right)=G\left(F_{s}\left(v\right)+h_{s}^{\left(l\right)}\left(v\right)\right)\text{,}$$

in which $m_{s}^{\left(l\right)}\left(v\right)$ remains to be a D-dimensional vector. Finally, the node's feature is updated using equation (A-4):

(A-4) $$h_{s}^{\left(l+1\right)}\left(v\right)=\sigma \left(m_{s}^{\left(l\right)}\left(v\right)\cdot W_{s}^{\left(l\right)}\right)\text{.}$$

After the static graph convolution completes the feature update for all nodes, the spatial topology structure of the entire graph remains unchanged.

Although the dynamic graph convolution calculation is also divided into three steps, it has evident differences from its static counterpart. Without loss of generality, we discuss the target node v at the l-th layer. For dynamic graph convolution, the local neighborhood $N_{d}\left(v\right)$ of each target node v is obtained by searching KNN in the current feature space with fixed parameter $k_{d}$. The specific form of the feature transformation function $F_{d}\left(\cdot \right)$ for message passing is given by equation (5) in subsection 3.4. In the local information aggregation stage, to take into account both the target node v itself and the information of its current neighborhood, a feature concatenation operation is required. The dynamic local neighborhood feature $m_{d}^{\left(l\right)}\left(v\right)$ is calculated using equation (6) in subsection 3.4. Finally, the node feature update stage can be simplified to a set of equation similar to (A-5):

(A-5) $$h_{d}^{\left(l+1\right)}\left(v\right)=\sigma \left(m_{d}^{\left(l\right)}\left(v\right)\cdot W_{d}^{\left(l\right)}\right)\text{.}$$

It is worth noting that the feature size of the network parameter matrix $W_{d}^{\left(l\right)}$ is 2D×D, whereas the corresponding $W_{s}^{\left(l\right)}$ in static graph convolution has a size of D×D.

### 2 The generalization ability of DBGCN on 3D datasets in other fields

Paris-Lille-3D is a benchmark dataset for street scene point cloud classification. We tested our DBGCN on Paris-Lille-3D dataset for evaluation on data generalization ability. The data was collected in two French cities (Paris and Lille) by a Mobile LiDAR System (MLS), including three street scene sub-datasets—the first is Lille1 street scene, 1150 m long with 71.3 million points (Lille1 is narrow in shape, and is split into two segments for storage); the second is Lille2 street scene, 340 m long with 26.8 million points; and the third is Paris street scene, 450 m long with 45.7 million points. All datasets have complete semantic point-level annotations for 10 classes. We selected a 630-m-long (30.0 million points) street scene from the first segment of Lille1 (see Fig. A2) for DBGCN testing and extracted two representative regions (Fig. A2(a1) and Fig. A2(b1)) for close view of the point-level classification performance. Fig. A2(a1) shows many parked vehicles along the street, while Fig. A2(b1) depicts several green vegetation areas with irregular boundaries.

We first applied FPS to reduce the number of points in both slices to 50,000, then randomly selected 2500 points as the training set $V_{GT}$ (5% annotation ratio) and the remaining 47,500 points as the test set $V_{raw}$. Figs. A2(a2) and **A2(b2)** show the training set distributions for the two slices, respectively. Due to the enormous size of the node input feature matrix X with 50000×50003, the computation speed can be quite slow; therefore, we employed PCA for dimensionality reduction on node feature X to reduce storage and speed up the process. The size of X after PCA is set to 50000×2048. DBGCN was initialized with l=4 and $k_{s}=k_{d}=30$, with hidden feature dimensions D=128. We trained for 300 epochs with a learning rate of 1e-3, followed by 800 epochs with a learning rate of 1e-4 on the best parameter model. Fig. A2(a3) shows label propagation results by DBGCN for the first slice, achieving 92.37% Acc and accurately segmenting different vehicles. Fig. A2(b3) shows label propagation results by DBGCN for the second slice, achieving 95.06% Acc. DBGCN precisely captured the boundaries of obstacles, vegetation, and ground categories in the second slice. In conclusion, the proposed DBGCN achieves satisfactory semi-supervised node inference on high-precision street scene point clouds, demonstrating its good generalization capability on 3D data outside the agriculture field.

## Appendix A. Supplementary data

Supplementary video related to this article can be found at https://doi.org/10.1016/j.plaphe.2026.100188

Multimedia component 1 An 8-minute video presentation of our work. Multimedia component 1
